# Canine melanomas: MiTF and p38 expression and its correlation with the presence of lymphocytic infiltrate, proliferation, and survival

**DOI:** 10.1007/s11259-026-11073-y

**Published:** 2026-01-28

**Authors:** Carlos Humberto da Costa Vieira-Filho, Jean Nunes dos Santos, Enio Ferreira, Karine Araújo Damasceno, Ricardo Wagner Portela, Vitor de Moraes Pina de Carvalho, Maria Carolina Santos Souza, Gilson Correia Carvalho, Alessandra Estrela-Lima

**Affiliations:** 1 Laboratório de Patologia Veterinária, Escola de Medicina Veterinária (EMEVZ), Universidade Federal da Bahia (UFBA), Salvador, 40170-110 Bahia State Brazil; 2 Laboratório de Patologia Cirúrgica Oral e Maxilofacial, Universidade Federal da Bahia (UFBA), Salvador, 40110-040 Bahia State Brazil; 3 Laboratório do Comportamento Celular, Instituto de Ciências Biológicas (ICB), Universidade Federal de Minas Gerais (UFMG), Belo Horizonte, 31270-901 Minas Gerais State Brazil; 4 Laboratório de Investigação em Saúde Global e Doenças Negligenciadas, Fundação Osvaldo Cruz (FIOCRUZ), Salvador, 40296-710 Bahia State Brazil; 5 Laboratório de Imunologia e Biologia Molecular, UFBA, Salvador, 40110-100 Bahia State Brazil; 6 Programa de Pós-Graduação em Ecologia, Instituto de Biologia, Instituto de Biologia (IBIo), UFBA, Salvador, 40170-110 Bahia State Brazil

**Keywords:** Carcinogenesis promoters, Cellular cycle, Dogs, Melanocytic neoplasias

## Abstract

The microphthalmia-associated transcription factor (MiTF) is considered a promoter of carcinogenesis in melanocytes and regulates several cellular processes. Its suppression in cell cultures is a consequence of the action of pro-inflammatory cytokines, and in rats, its downregulation occurs via the p38 pathway. This study aimed to evaluate the expression of MiTF and p38 in canine melanomas using immunohistochemical assays and to verify the correlations between this expression and the presence of an inflammatory infiltrate, tumor size, mitotic index, and animal survival. One hundred seventeen samples of canine melanomas from the oral cavity/digits (*n* = 74) and skin (*n* = 43) were analyzed. In oral/digit melanomas, positive relationships were observed between larger tumors (*p* = 0.007), higher mitotic index, and the absence of MiTF expression. In cutaneous melanomas, MiTF expression was correlated with a greater number of tumors, whereas p38 expression was associated with smaller tumors, the presence of a lymphocytic infiltrate, and prolonged survival. We infer that the expression of MiTF and p38 is higher in canine skin tumors and that this expression may be related to reduced aggressiveness of these neoplasias and less tumor progression. Interestingly, the reduced MiTF expression observed in larger and more mitotically active tumors, such as oral/digit melanomas, appears to contrast with its known role in promoting carcinogenesis. However, this reduction may reflect dedifferentiation and increased aggressiveness of neoplastic melanocytes.

## Introduction

The expression of cell cycle regulatory proteins has been studied in human melanoma cell lines (Aguirre-Ghiso et al. 2001) and animal models (Noguchi et al. 2016). Among these proteins, the microphthalmia-associated transcription factor (MiTF) (Trivedi et al. 2020) and p38 (Aguirre-Ghiso et al. 2001) stand out. MiTF is a master regulator of melanocyte homeostasis (Hartman and Czyz 2015). However, its complex role in carcinogenesis involves oncogenic and tumor-suppressor pathways (Pilch et al. 2025). Its expression is maintained in most melanomas and is observed in metastatic lesions (King et al. 2001). It is believed that the primary functions of MiTF in melanoma include maintaining cell line identity by controlling cell differentiation markers and playing an anti-apoptotic role (Vlčková et al. 2018). In rats, the p38 pathway activates control of MiTF expression, which inhibits cell growth (Smalley and Eisen 2000) and induces neoplastic cell senescence (Zhang et al. 2013). Interestingly, this event does not occur in some human melanomas due to the regulation of apoptosis by other pathways unrelated to p38 (Ivanov and Ronai 2000). The immunohistochemical (IHC) expression of MiTF remains controversial regarding its specificity and sensitivity. In a study on an IHC panel for the diagnosis of melanocytic neoplasms in dogs (Smedley et al. 2011), MiTF was observed to have 20% specificity and 91.80% sensitivity in these tumors, as well as cytoplasmic staining in some cases. Differences in staining with immunofluorescence and IHC were also observed (Campagne et al. 2013).

Human cutaneous melanomas elicit a significant anti-tumor response that is primarily mediated by lymphocytes. This response is considered an independent prognostic factor (Sinnamon et al. 2018). Low MiTF expression has been correlated with increased cytokine production and a more invasive, undifferentiated phenotype (Reinhardt et al. 2017; Huang et al. 2010). These tumor-infiltrating lymphocytes (TILs) are associated with a better prognosis with superficial tumors and/or those in a regression stage (Clark et al. 1989). Studies have attempted to characterize TILs in dogs and their importance in canine melanomas, reporting differences related to lymphocyte subpopulations and their association with prognostic factors and survival rates. For example, higher numbers of CD3 + T cells (Buffon et al. 2022) and CD20 + cells (Porcellato et al. 2020) have been associated with related factors and worse prognoses. FoxP3 + cells have been associated with higher Cox-2 expression and more aggressive tumors (Silveira et al. 2020). The presence of TILs, particularly with a higher proportion of CD8 + cells, has been linked to improved survival (Yasumaru et al. 2021). These findings underscore the complex biological dynamics that influence the progression and prognosis of canine melanoma, highlighting the need for a deeper understanding of its molecular mechanisms.

Melanomas are common in dogs, mainly affecting oral (62%), cutaneous (27%), and digital/subungual (10%) sites (Polton et al. 2024). Only non-UV-induced cutaneous and mucosal melanomas are considered comparative models for human disease due to shared etiology. Cutaneous melanomas exhibit less aggressive behavior and better prognosis than oral forms, while digital melanomas are a subset of cutaneous ones (Smedley et al. 2011; Silvestri et al. 2019). Given the relevance and high frequency of melanomas in dogs, the objective of this study was to evaluate the expression of MiTF and p38 in canine melanomas and to investigate the correlations of this expression with the presence of a lymphocytic infiltrate, prognostic factors, and the survival of dogs bearing melanomas in different sites.

## Materials and methods

This study was carried out in accordance with the ethical principles for the use of animals in experimentation. It was approved by the Ethics Committee on the Use of Animals (CETEA/CEUA) of the School of Veterinary Medicine of the Federal University of Bahia (Protocol number 010/2018).

One hundred seventeen melanoma samples (oral cavity/digits or skin) from incisional and excisional biopsies, obtained from dogs of different breeds, were sent to the Laboratory of Veterinary Pathology of the Federal University of Bahia and analyzed. Consistent with established prognostic factors that emphasize anatomical location as a significant determinant of biological behavior in canine melanoma (Polton et al. 2024), the selected cases were divided into two groups: the first consisted of animals with aggressive melanomas located in the oral cavity or digits (*n* = 74), and the second consisted of animals with cutaneous melanoma (*n* = 43), which in most exhibited lower aggressiveness. The lesions were grouped based on their historically more aggressive biological behavior (oral and digital) in comparison to other cutaneous sites (Smedley et al. 2011; Polton et al. 2024). Only digital lesions displaying histological parameters on H&E sections associated with a poorer prognosis (invasion extent, low melanin production, ulceration, and mitotic index) were included in the aggressive group.

The histological samples of canine melanomas were fixed in neutral formaldehyde buffered with 10% phosphate (monobasic and dibasic), included in paraffin, stained using the hematoxylin-eosin technique, and independently analyzed under optical microscopy by two veterinary pathologists. Cases with diagnoses confirmed by the two evaluators were included in the study. In the microscopic evaluation, prognostically significant parameters such as tumor thickness and mitotic index were determined, along with the association of lymphocytic inflammatory infiltrate with neoplasia, consistent with recognized histological features for canine melanoma assessment (Polton et al. 2024). All evaluations were performed using an optical microscope (ICC50, Leica, Wetzlar, Germany) equipped with a 10× eyepiece and a 40× objective, which provided a field area of 0.239 mm².

The tumors were segregated according to thickness (< 0.95 cm or ≥ 0.95 cm), using a conventional ocular ruler (Silvestri et al. 2019). Incisional biopsies were included only for oral melanomas with depths greater than 2.0 cm, in which complete excision was anatomically limited. Tumor thickness was measured from the epithelial junction to the deepest visible neoplastic cells. All cutaneous melanomas were obtained through complete excisional biopsies, allowing full margin and depth evaluation. The inclusion of tumor thickness as a variable was maintained due to its biological relevance and the potential for digital lesions to exhibit greater aggressiveness. For the mitotic index, mitoses were identified by observing ten fields, excluding those with necrosis or artifacts, using a 40× objective and a 10× eyepiece, with a field of view of 22 mm in diameter and a level field of 0.55 mm in each sample. Each field had an area of 0.237 mm², and the average per case was obtained (high-average equal to or greater than three and four mitoses per field in cutaneous and oral/digit tumors, respectively). The morphological analysis of the inflammatory reaction associated with the tumor was performed in terms of its presence and distribution (Clark et al. 1989).

**Fig. 1:**
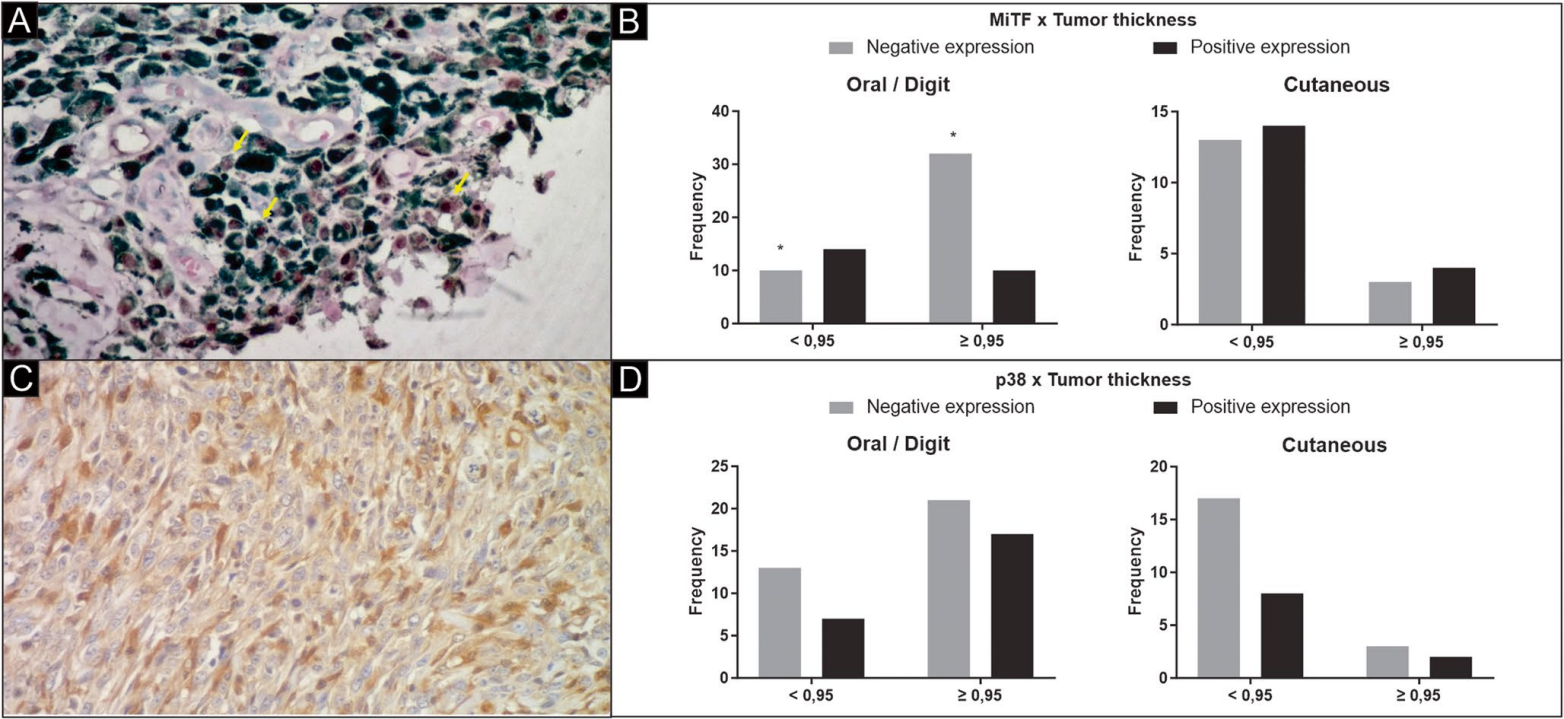
Expression of MiTF and p38 in canine melanomas, as defined by immunohistochemistry. (**A**) Canine melanoma with nuclear immunohistochemical immunostaining (arrows) in neoplastic melanocytes for anti-MiTF antibody; magnification 400×. (**B**) Comparison of MiTF expression in canine melanomas with thicknesses < or ≥ 0.95 cm located in the oral cavity/digits and on the skin. Bottom) (**C**) Cytoplasmic immunostaining in neoplastic melanocytes for anti-p38 antibody; magnification 400×. (**D**) Comparison of p38 expression in canine melanomas with thicknesses < or ≥ 0.95 cm located in the oral cavity/digits and on the skin. * Significant difference *p* < 0.05 in Fisher’s test

Immunohistochemistry was performed using the peroxidase reaction technique with detection through a polymerized secondary antibody (Novolink Polymer Detection System, Leica Biosystems, Newcastle upon Tyne, UK; and Histofine Simple Stain MAX PO MULTI, Nicherei Fresh, Tokyo, Japan). Antigenic recovery was achieved by pressurized wet heat at 125 °C (Pascal Pressure Cooker, Dako Cytomation, Glostrup, Denmark) using a Target Retrieval Citrate Solution (pH 6.0, Dako Cytomation). To block endogenous peroxidase, the slides were incubated for two 10-minute periods in a solution of 3% H_2_O_2_ in methyl alcohol. The slides were incubated for 20 min to block endogenous proteins in Protein Block Serum-Free Ready-to-Use solution (Dako North America, Via Real Carpinteria, CA). The reagents were applied by a manual technique, with a primary antibody incubation time of 30 min. The anti-MiTF antibody was diluted 1:60 (Abcam, Cambridge, UK), and the anti-p38 MAPK antibody was diluted 1:100 (Abcam). A further incubation with chromogen 3’3-diaminobenzidine (Liquid DAB + Substrate Chromogen system, Dako North America) was conducted for one minute. After developing with DAB, the cuts were counterstained with Giemsa (1:5) for 10 min and then washed in a hydrochloric acid solution (1:100), followed by absolute alcohol and finally isopropyl alcohol. With this treatment, the melanin pigment acquired a greenish tint after counterstaining, allowing for the visualization of the brownish chromogenic reaction with DAB. Positive controls consisted of normal canine skin containing epidermal and follicular melanocytes, known to express MITF, and mammary carcinoma for p38. Negative controls were obtained by replacing the primary antibody with PBS. For the analysis of the immunomarking by the anti-MiTF and p38MAPK antibodies, after the identification and marking of the fields (“hotspots”) in each case, the reaction was considered significant (nuclear and nuclear/cytoplasmic, respectively), regardless of the intensity of the same (Fig. 1A and C). The evaluation was made based on the counting of immunostained cells and classified into scores of 0 (missing mark) and 1 (present mark) (Shah et al. 2010). To assess the agreement between the two pathologists involved in the histopathological analysis and the assignment of IHC, the Kappa concordance coefficient was calculated, yielding a value of 0.90.

Consultations were conducted with the veterinarians who requested the histopathological examinations and the animals’ clinical records, from which data on overall survival in days were collected. Cases in which it was not possible to obtain this information and/or when the patient died due to causes unrelated to the melanoma were not included in the analysis of survival.

The data distributions were evaluated using the Kolmogorov–Smirnov test, and parametric or nonparametric statistical tests were selected accordingly. For comparisons between two groups, Student’s t-tests (for normally distributed data) or Mann–Whitney U tests (for non-normal data) were conducted. For comparisons involving more than two groups, one-way analyses of variance (ANOVAs) were performed for parametric analysis, or Kruskal–Wallis tests were used for nonparametric analysis. For all ANOVAs, the assumptions of normality of residuals, homogeneity of variances, and independence of observations were evaluated. Categorical variables were analyzed using Fisher’s exact test. Correlations were tested using Pearson’s (parametric) or Spearman’s (nonparametric) correlation coefficients. Survival curves were estimated using the Kaplan–Meier method and compared using the log-rank (Mantel–Cox) test. Statistical significance was set at *p* < 0.05 for all analyses. The analyses were conducted using Prism 5.0 (GraphPad, San Diego, CA) and SPSS 17 (SPSS Inc., Chicago, IL).

## Result

Immunohistochemically, MiTF showed nuclear labeling in neoplastic melanocytes, with moderate to strong intensity in cutaneous melanomas and weak or absent staining in most oral and digital lesions. p38 expression was cytoplasmic and diffuse, showing moderate intensity in cutaneous melanomas and reduced or absent labeling in aggressive tumors. Intratumoral heterogeneity was observed, with stronger immunoreactivity in less pigmented regions.

**Fig. 2:**
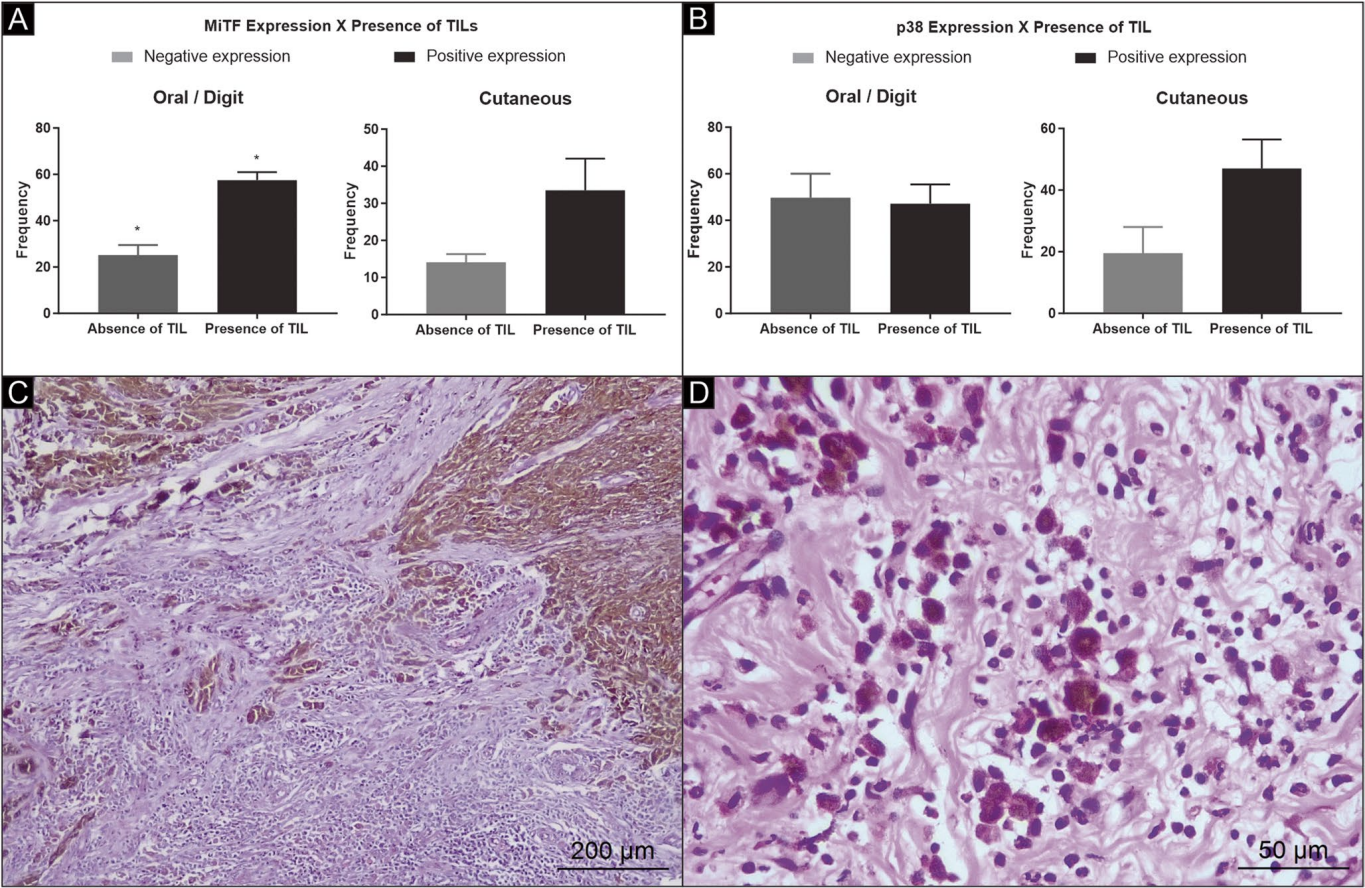
Association between MiTF and p38 expression and tumor-infiltrating lymphocytes (TILs) in canine melanomas. (**A**) Association between MiTF expression and the presence of TILs in cutaneous and oral/digital melanomas; (**B**) Association between p38 expression and the presence of TILs in the same anatomical sites. Photomicrographs staining in H&E of a canine melanoma exhibiting marked inflammatory infiltrate intra/peritumoral multifocal: (**C**) 100 × magnification; Highlight C, showing predominantly lymphocytic intratumoral inflammatory infiltrate. (**D**) 400 × magnification. * Statistical significance: *p* < 0.05 (Fisher’s exact test)

The immunohistochemical analysis revealed a positive association between the expression of MiTF and p38 protein (*p* = 0.0268). A higher expression of MiTF was observed in skin melanomas, compared with those in the oral cavity or digits (*p* = 0.0347). The lesions in the digits with thickness ≥ 0.95 cm presented a lower expression of MiTF (*p* = 0.0077). Skin tumors showed a greater expression of MiTF regardless of the dimensions and thickness of the lesion (Fig. 1B). The non-expression of MiTF was related to a high mitotic index in tumors of the oral cavity/digits. The MiTF expression was correlated with the lymphocytic inflammatory response, regardless of its intensity and location (*p* = 0.0004) (Fig. 2A).

The immunomarking of the p38 protein revealed a greater expression in melanomas located on the skin (*p* = 0.0913) (Fig. 1D), when compared to melanomas of the oral cavity/digits, although no statistically significant difference was observed. Most lesions in the oral cavity/digits smaller than 2.0 cm or with a thickness < 0.95 cm revealed p38 expression. Skin lesions showed expression of this protein regardless of nodule size and thickness. The expression of p38 was similar in melanomas located in the oral cavity/digits (*p* = 0.9188) without or with a marked inflammatory response associated (Fig. 2C and D).

**Fig. 3:**
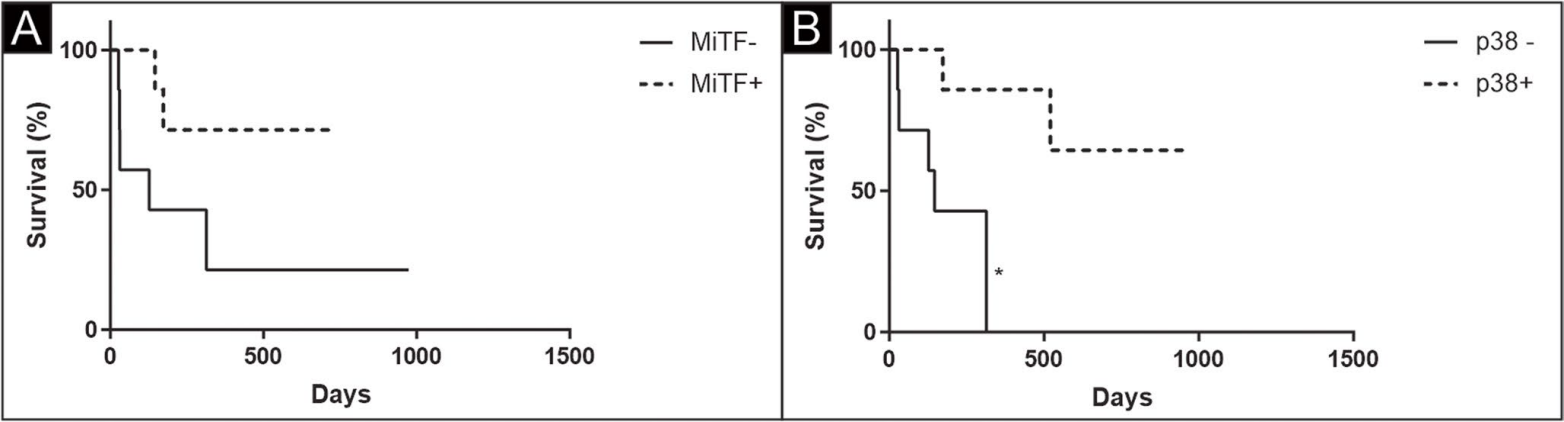
Survival curves stratified by the expression of MiTF and p38, regardless of the location of the melanoma (oral cavity/digits or cutaneous). Survival curves were estimated using the Kaplan-Meier method, followed by the Log-rank test. *Significant difference (*p* = 0.0152)

In cutaneous tumors, this protein was more expressed in melanomas when this infiltrate was present (*p* = 0.2885) (Fig. 2B). To assess survival, information was obtained on 17 animals (10 with oral/digit melanomas and 7 with cutaneous melanomas). More deaths were observed in the group of animals with tumors that did not express p38 (*p* = 0.0152; median survival time, 146 days) and MiTF (*p* = 0.0828; median survival time, 127 days) (Fig. 3). The minimum survival after surgery was 27 days (male, Dachshund breed, melanoma in the oral cavity, ≥ 0.95 cm), and the longest survival, within the study interval, was 1169 days (male, Rottweiler breed, melanoma in the digit, < 0.95 cm). The median survival was 136 days.

## Discussion

Evaluation of the expression of MiTF and p38 is crucial for understanding the biology of neoplastic melanocytes, as well as their differentiation and proliferation (Ploper and De Robertis 2015). Cells with low MiTF activity are associated with a more aggressive cell phenotype (Hartman and Czyz 2015), while p38 is associated with the promotion of melanogenesis and inhibition of cell growth (Smalley and Eisen 2000). The present study evaluated the expression of MiTF and p38 proteins in melanomas located in different canine anatomical sites.

MiTF displays context-dependent behavior during melanoma progression. King et al. (2001) reported its upregulation in metastatic lesions, consistent with its role in proliferation and survival. Other studies have shown that reduced MiTF activity can promote dedifferentiation and invasiveness (Hartman and Czyz 2015). This apparent duality reflects the dynamic “phenotype switching” characteristic of melanoma biology.

Most of the cutaneous melanomas analyzed in this study expressed MiTF and p38. These results can be attributed to the greater differentiation, smaller dimensions, and possibly lower aggressiveness of these skin tumors (Nishiya et al. 2016, Stevenson et al. 2023). In contrast, melanomas located in the oral cavity/digits showed less expression of these proteins in larger tumors with a high mitotic index, as well as in primary lesions with metastatic foci. These results suggest that the more melanomas express MiTF and p38, the longer these animals survive.

Although previous studies have shown near-universal MiTF nuclear positivity by immunofluorescence (Campagne et al. 2013), immunohistochemical analyses tend to reveal variable sensitivity and specificity (Smedley et al. 2011). The use of Giemsa counterstaining after DAB minimized pigment interference, allowing accurate distinction of specific labeling. The occasional cytoplasmic expression observed here is consistent with previous findings. Therefore, the lower MiTF expression detected by IHC may reflect reduced differentiation in biologically more aggressive oral and digital melanomas. The survival results reported herein should be considered exploratory because of the limited number of cases examined.

MiTF expression was also found to be correlated with the presence of TILs in tumors of the oral cavity and digits, regardless of the lesion’s location. This finding indicates that these lesions exhibit greater cell differentiation and a capacity to elicit an inflammatory response (Zhang et al. 2013). However, this lymphocytic infiltrate is not necessarily composed of activated cells or cells with anti-tumor activity (Ladányi et al. 2011), given that pro-inflammatory cytokines are associated with the suppression of MiTF and the chemotaxis of myeloid cells to the tumor site (Reinhardt et al. 2017).

Even in the cases with the highest MiTF expression associated with TILs, oral and digital melanomas did not exhibit more aggressive behavior. In fact, immunohistochemical analyses performed in some of these cases revealed a predominance of CD4 + and FoxP3 + cells in these aggressive tumors (data not shown), suggesting an immunosuppressive microenvironment. This finding aligns with the prognostic implications described by Porcellato et al. (2020) and warrants further investigation in larger cohorts.

The expression of p38 is associated with the induction of apoptosis and the senescence of neoplastic cells (Zhang et al. 2013). Like MiTF, p38 had greater expression in tumors of smaller dimensions/thickness, regardless of the evaluated group (oral/digit, or cutaneous). This situation suggests growth suppression in these tumors, characterized by smaller dimensions and less aggressive biological behavior (Smalley and Eisen 2000).

The p38 MAPK pathway exerts antiproliferative effects and plays a positive role in melanogenesis by activating α-MSH, which stimulates melanocyte differentiation. The influence of α-MSH on cell proliferation remains controversial, with both proliferative and antiproliferative effects reported, suggesting that additional regulatory factors are involved. In poorly differentiated or weakly pigmented melanomas, loss of α-MSH/p38 activity may compromise its antiproliferative function. p38 primarily inhibits cell growth in non-neoplastic cells, and isolated p38 inhibition can interfere with melanocytic differentiation (Smalley and Eisen 2000; Aguirre-Ghiso et al. 2001).

Although MiTF has been described as a carcinogenesis promoter, its biological role in melanoma is complex and context-dependent. MiTF acts as a key regulator of melanocyte differentiation and survival; however, low MiTF activity has been associated with increased invasiveness, loss of differentiation, and activation of alternative proliferative pathways, a process known as phenotype switching (Hartman and Czyz 2015; Ploper and De Robertis 2015; Reinhardt et al. 2017; Vlčková et al. 2018). This complex interplay is robustly demonstrated in human uveal melanoma, where Pilch et al. (2025) identified distinct “MiTF-low” tumors characterized by slow proliferation, high invasive potential, and an inflammatory phenotype, with loss of BAP1, a key tumor suppressor gene, contrasting with proliferative “MiTF-high” tumors that exhibit lower invasiveness. Therefore, the reduced MiTF expression observed in the more aggressive canine melanomas in this study likely reflects this dedifferentiated state and enhanced tumor plasticity, rather than contradicting its oncogenic role.

The absence of TILs was also observed in oral tumors/digits that do not express the p38 protein. The opposite was observed in skin tumors: most tumors associated with TILs expressed the p38 protein. The greater expression of p38 is associated with a lymphocytic response, which is related to the suppression of tumor growth resulting from increased production of TNF by inflammatory cells (Huang et al. 2010). Although this association has not been demonstrated in dogs, previous studies in other species suggest that MiTF and p38 may be linked to TNF-mediated inflammatory pathways, and this remains a speculative consideration in the present study.

Thus, it is believed that the expression of p38 in oral/digit tumors without an inflammatory response may not be effective as one of the mechanisms for the control of the cell cycle and not exert its suppressive action in the signaling of RAS or activation of other pathways that lead to proliferation (Ivanov and Ronai 2000). Additionally, the possibility that TILs associated with larger tumors may become anergic or senescent, leading to the loss of their anti-tumor effects, must be considered (Hussein et al. 2006).

These results confirm the expression of MiTF and p38 as potential prognostic factors in canine melanomas, as the absence of expression of these molecules was associated with a higher mortality rate and shorter survival. In addition, the expression of MiTF and p38 is higher in skin tumors, indicating that in smaller and well-differentiated tumors, this expression is associated with less tumor aggressiveness. In oral/digit melanomas, the non-expression of MiTF and p38 is correlated with clinical-pathological characteristics associated with a worse prognosis, supporting the idea that less differentiated neoplasms exhibit less activation of apoptotic pathways.

King et al. (2001) reported MiTF positivity in benign and malignant melanocytic proliferations, albeit with reduced staining intensity or loss in some malignant cases. This variability is fundamental and was further elucidated by Pilch et al. (2025) in uveal melanoma, who described a dualistic and context-dependent role for MiTF. They identified two distinct tumor subpopulations: tumors with low MiTF expression (“MiTF-low”) exhibited a more aggressive phenotype, significantly correlating with distant metastases, BAP1 loss, larger tumor diameter, and an inflammatory response mediated by tumor-associated macrophages (TAMs), resulting in shorter survival. In contrast, tumors with high MiTF expression (“MiTF-high”) demonstrated lower invasiveness, associated with better clinical prognoses but greater cellular proliferation. While our findings corroborate that higher MiTF expression is associated with prolonged survival and reduced tumor aggressiveness, it is fundamental to note, as per Pilch et al. (2025), that MiTF expression alone did not prove to be an independent prognostic factor in multivariate analyses, with its prognostic significance being contextualized by other established markers. This nuance underscores the complexity of MiTF as a biomarker and a potential therapeutic target.

Although MiTF expression appeared to be reduced in aggressive oral and digital melanomas, this finding may reflect both the technical limitations of IHC in heavily pigmented lesions and actual biological differences related to dedifferentiation. IHC is a more practical and more widely used diagnostic method than immunofluorescence, and its lower sensitivity could partially explain the reduced detection. However, this reduced expression may also represent a less differentiated cellular phenotype typical of biologically aggressive melanomas.

This study had some limitations stemming from its design. The survival analysis was conducted on a relatively small cohort and was retrospective in nature, which may affect the statistical robustness and generalizability of specific prognostic associations. Furthermore, the characterization of tumor-infiltrating lymphocytes primarily focused on their presence and distribution, lacking detailed phenotyping of immune subpopulations, which precluded a more in-depth understanding of the precise role of these cells in disease progression. These limitations do not diminish the value of the findings presented herein but do identify important directions for future investigations involving larger cohorts and more comprehensive approaches.

## Data Availability

The original contributions presented in this study are included in the article/Supplementary material; further inquiries can be directed to the corresponding authors.
